# Reimagining Connected Care in the Era of Digital Medicine

**DOI:** 10.2196/34483

**Published:** 2022-04-15

**Authors:** Devin M Mann, Katharine Lawrence

**Affiliations:** 1 Department of Population Health NYU Grossman School of Medicine New York, NY United States; 2 Medical Center Information Technology NYU Langone Health New York, NY United States

**Keywords:** health information technology, telehealth, remote patient monitoring, mobile health, mHealth, eHealth, digital health, innovation, process model, information technology, digital medicine, automation

## Abstract

The COVID-19 pandemic accelerated the adoption of remote patient monitoring technology, which offers exciting opportunities for expanded connected care at a distance. However, while the mode of clinicians’ interactions with patients and their health data has transformed, the larger framework of how we deliver care is still driven by a model of episodic care that does not facilitate this new frontier. Fully realizing a transformation to a system of continuous connected care augmented by remote monitoring technology will require a shift in clinicians’ and health systems’ approach to care delivery technology and its associated data volume and complexity. In this article, we present a solution that organizes and optimizes the interaction of automated technologies with human oversight, allowing for the maximal use of data-rich tools while preserving the pieces of medical care considered uniquely human. We review implications of this “augmented continuous connected care” model of remote patient monitoring for clinical practice and offer human-centered design-informed next steps to encourage innovation around these important issues.

## The Growth of Remote Patient Monitoring: Accelerating the Transition From Episodic to Continuous Care

The COVID-19 pandemic accelerated the adoption of remote patient monitoring (RPM)—the use of ambulatory, noninvasive digital technology to capture and transmit patient data in real time for care delivery and disease management. The use of RPM technology during the pandemic has allowed for care at a distance in an age of unprecedented health uncertainty and disruption [1]. However, while the mode of clinicians’ interactions with patients and their health data has transformed, the larger framework of *how we deliver care* has only incrementally shifted. Most health care is still delivered in episodes—synchronous moments of connection between clinicians and patients, mediated by discrete hospitalizations, office visits, or video and audio calls. But health is *not* episodic; it is a fundamental part of the human condition, experienced regularly and continuously, more akin to a utility (eg, energy, water, or even education) than a traditional professional service (eg, tax preparation). The friction in these competing visions of care has contributed to a fragmented, inconsistent health care delivery experience; it has also limited the health information technology resources, innovations, and capital needed to make the world of data-driven continuous care possible. RPM technologies have the potential to enable and accelerate a transition from episodic to continuous care. Here, we outline the current state of RPM, its challenges in clinical practice, and how a continuous connected care model can be organized based on technology-driven transitions that include not only RPM but also the larger world of digital health technologies (eg, telehealth, machine learning, and artificial intelligence [AI]).

## The Tidal Wave of RPM Data

The rapid adoption of new digital health care technologies for remote health care provision has begun to dismantle the barrier between the clinic and the home. The influx of health data from RPM devices has the potential to realize a new framework of medicine—not just of higher quality episodic medicine but of a continuous connected care model that reflects the true interaction of health care and the human experience. However, while more continuous and complete data from patients can facilitate this new world of care, its introduction into the health care landscape has also raised concerns [2]. RPM devices generate more data than a clinician is equipped to manage, and current health information technology systems are inadequate for the data curation and visualization required to effectively use these data to help patients [3]. At the same time, there are worries that simply increasing the *volume* of data about a patient does not translate to improved health outcomes, and that more understanding of the *quality* requirements of RPM is needed [4]. Finally, the increased burden on patients and clinicians to *always be connected*—whether to their health or their jobs—has raised concerns about medical overutilization, burnout, and excessive consumerism; even the term “remote patient monitoring” is problematic, conjuring images of invasive surveillance and control rather than supportive care [5,6].

## RPM and the Augmented Continuous Connected Care Pyramid

To address these challenges, health systems have taken several approaches to managing RPM in clinical practice; these include limiting digital access and services for patients, adding new staff, or implementing automated tools such as chatbots and AI models to manage the data and their clinical consequences [7,8]. Digital health vendors are increasingly offering (and payors are reimbursing) digital and virtual health services that include RPM devices with improving interoperability (leveraging standard interfaces) among vendors and electronic health records [9-11]. These new platforms enable more robust acute, chronic, and home hospital management [12-15]. Other digital health tools have been implemented that use AI to evaluate RPM data inputs and help boost the important signals buried within the noise. For example, DreaMed uses AI to sift through the mountains of RPM-generated continuous glucose monitoring and insulin pump data to make specific recommendations for insulin titrations [16]. Clinical staff are increasingly being tasked with managing the incoming RPM data streams and supporting providers in delivering a more continuous connected care experience. Ochsner’s RPM driven hypertension program is a successful example of this new paradigm in practice [17,18]. Ultimately, a solution that optimizes the interaction of automated technologies with human oversight, both routine and specialized, will likely prevail, allowing for maximal use of data-rich technologies while preserving the pieces of medical care considered “uniquely” human.

**Figure 1:**
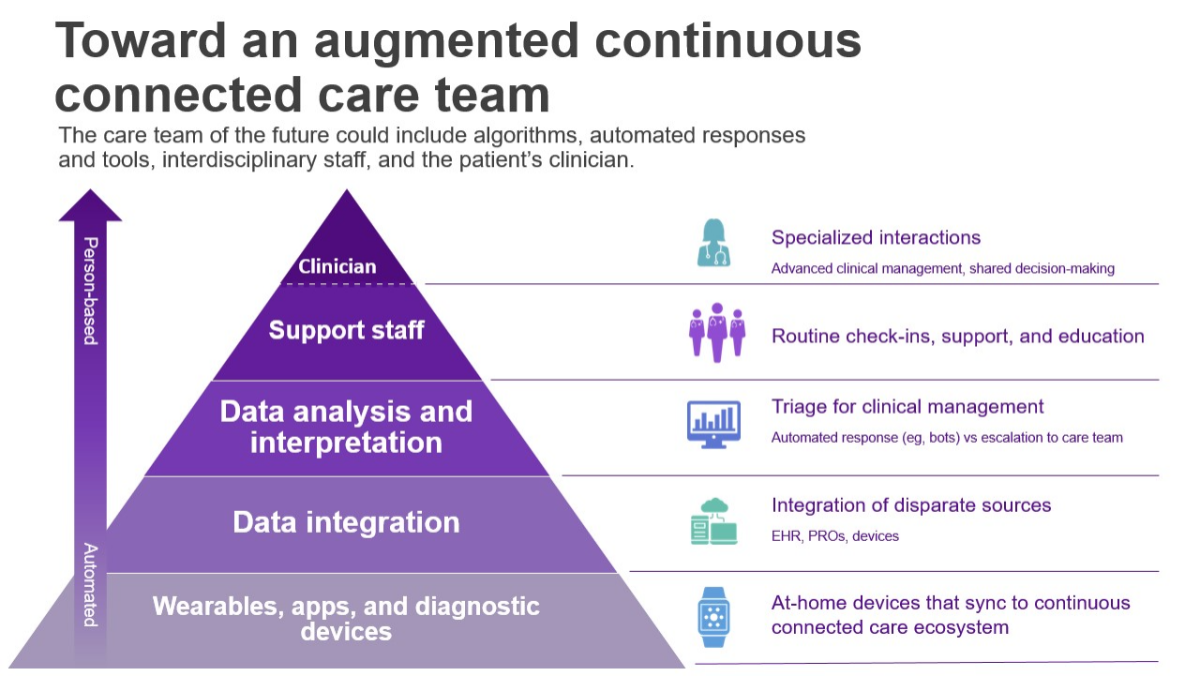
Augmented continuous connected care pyramid. EHR: electronic health record; PRO: patient-reported outcomes.

Taken together, RPM tools and their implementation in health care systems can be viewed as an example of a novel pyramid of health care delivery, “augmented continuous connected care” (Figure 1). At the base, continuous connected care is built on an always “on,” automated, and often passive, holistic health data capture layer. These diverse data points are integrated and standardized by an algorithmic (or a machine learning) layer that can “listen” to and interpret data and either respond autonomously or reduce noise and boost signals to generate more actionable insights for human interpretation. Decisions beyond the scope of this “digital clinician” are routed to the clinical team for management, enabling them to work to the top of their licenses and provide the parts of medical care that benefit most from a human touch—patient education, shared decision-making, and complex medical decision-making.

The benefits of RPM-enabled connected continuous care continue to emerge as the pandemic progresses and health care systems respond and evolve. Technologies that were once considered niche products for rural health or areas with resource or accessibility barriers have proven translatable to a variety of health care settings and contexts, enabling diverse populations to manage both acute and chronic conditions with improved information, safety, and convenience [19,20]. Shared management of RPM data, particularly after AI processing, is a more sustainable model for supporting continuous care and allows patients greater connectedness with their health care team.

## Using Design Thinking to Identify and Overcome Challenges to RPM-Enabled Continuous Care

The impact of health care’s shift to an augmented continuous connected care paradigm will be significant for patients, providers, and care delivery systems. Below, we frame key implications as a set of Design Thinking–informed “how might we” questions to encourage new thinking around these issues [21]. These questions are by no means exhaustive of the challenges facing the future of continuous connected care, but are designed to inspire health care leaders, designers, and clinicians to reimagine these and other key concerns related to RPM and the broader world of digital health technology.

1. How might we use RPM technologies to let patients easily share their “daily life data” without overwhelming their health care teams?
2. How might we change how patients and health care teams communicate to take advantage of continuous connected care?
3. How might we transform the health care workforce to manage this new type of high volume, daily life data?

These questions acknowledge both the potential and the challenges of using RPM and other digital health tools to evolve the world of health care. The answers to these questions require improvements in the design, usability, and interoperability of RPM tools to reduce the burden of patient work and empower equitable access to and use of this transformative technology. It will also require careful balancing of the benefits of continuous care with the risks of being “too connected.” New approaches will be needed that help patients and clinicians stay updated on information and make actionable decisions on the key events in their health while also protecting themselves and their data.

## Implications of Augmented Continuous Connected Care for Practitioners, Researchers, and Policy Makers

The rapid growth of RPM and the continuous connected care paradigm it enables requires new research and development to improve the devices, the platforms, integrated AI, interoperability, and the usability of RPM tools. New team structures, personnel, and workflows will need to be identified, tested, and disseminated to help health care systems and patients take full advantage of this enabling technology. Clinicians will need better EHR-integrated tools, training, and team-based support to manage patients using a mix of clinic and home-based data collection and leverage AI-based tools to highlight critical insights and actions. To support these new workflows, clinical delivery system leaders will need organizational and regulatory flexibility to modify health care workers’ composition and scopes of work; such support may require investment in new roles such as RPM “navigators,” community health workers, and others who manage data and devices in partnership with patients and clinicians. Policy makers will need to foster a regulatory environment and financial incentives that incentivizes RPM innovation while shepherding the industry to adhere to common standards and the free flow of data across systems.

RPM and other digital health technologies are ushering in a transition to a new and exciting model of care, from episodic to continuous and connected. However, there are many challenges facing this transition, and how we respond to them will shape the ultimate impact of not only these tools themselves but also our ability to use these technologies to improve health care experiences and outcomes.

## Abbreviations

AI: artificial intelligence
RPM: remote patient monitoring
